# HOUSING, AGING, AND HEALTH: NEW FINDINGS AND FRAMEWORKS FROM HOUSING-FOCUSED RESEARCH IN THE CONTEXT OF COVID-19

**DOI:** 10.1093/geroni/igac059.1329

**Published:** 2022-12-20

**Authors:** Nancy Berlinger

## Abstract

Older adults have been the most vulnerable population to severe illness, hospitalization, or death from COVID-19. During the pandemic, housing became a site of health and safety for some, while reinforced inequities for others due to underlying problems of affordability, accessibility, safety, and service access. This symposium showcases five housing-focused studies reflecting the pandemic context, including research by early-career investigators. The first speaker will present findings from a study of materials preserving promising practices and policy ideas generated by housing-focused pandemic responses to middle-income and low-income community-dwelling older adults. The second speaker will discuss narrative lessons from “avoidance hotels” – an infection-reduction strategy that transferred older adults from shelters to hotel rooms – with potential to guide planning for the needs of older adults facing homelessness and serious illness. The third speaker will analyze findings from multi-city research on the pandemic housing experiences of lower-income Black women, who faced severe intersectional threats to housing security. The fourth speaker will share data from a qualitative study of a Medicare-financed home care program for residents of HUD housing, including recommendations for improving experiences of participants and providers. The fifth speaker will describe the evolution of the pandemic-response roles of HUD service coordinators, based on findings from surveys in 2020 and 2021. We will conclude with audience discussion about ways for age-focused researchers to collaborate in crafting policy solutions and effective public narratives about housing equity in America's aging society.

